# Phenotype Definition for “Resisters” to *Mycobacterium tuberculosis* Infection in the Literature—A Review and Recommendations

**DOI:** 10.3389/fimmu.2021.619988

**Published:** 2021-02-25

**Authors:** Jesús Gutierrez, Elouise E. Kroon, Marlo Möller, Catherine M. Stein

**Affiliations:** ^1^ Department of Population and Quantitative Health Science, Case Western Reserve University School of Medicine, Cleveland, OH, United States; ^2^ DSI-NRF Centre of Excellence for Biomedical Tuberculosis Research, South African Medical Research Council Centre for Tuberculosis Research, Division of Molecular Biology and Human Genetics, Faculty of Medicine and Health Sciences, Stellenbosch University, Cape Town, South Africa

**Keywords:** tuberculosis, resister, phenotype, tuberculin skin test, interferon-γ release assay, review

## Abstract

Tuberculosis (TB) remains a worldwide problem. Despite the high disease rate, not all who are infected with *Mycobacterium Tuberculosis* (*Mtb*) develop disease. Interferon-γ (IFN-γ) specific T cell immune assays such as Quantiferon and Elispot, as well as a skin hypersensitivity test, known as a tuberculin skin test, are widely used to infer infection. These assays measure immune conversion in response to *Mtb*. Some individuals measure persistently negative to immune conversion, despite high and prolonged exposure to *Mtb*. Increasing interest into this phenotype has led to multiple publications describing various aspects of these responses. However, there is a lack of a unified “resister” definition. A universal definition will improve cross study data comparisons and assist with future study design and planning. We review the current literature describing this phenotype and make recommendations for future studies.

## Introduction

Tuberculosis (TB) remains a major public health problem globally. Since 2007, *Mycobacterium tuberculosis* (*Mtb*) has been responsible for the greatest number of deaths from a single infectious agent around the world. In 2018, 10.0 million people developed active disease and approximately 1.5 million people died (1). One of the challenges faced in developing effective TB preventative treatment strategies is understanding the underlying innate and adaptive responses to natural clearance of *Mtb.* We currently lack a gold standard to measure infection and can only infer it from a tuberculin skin test (TST) or *in vitro* based Interferon-γ (IFN-γ) release assays (IGRA). These assays are markers of immune conversion in response to *Mtb* exposure and do not capture the full spectrum of anti-*Mtb* immunity (2–4). Despite this inherent limitation, the study of persons who test persistently negative for these assays and do not develop TB provide unique and important epidemiological, genetic, and immunological insights into understanding the possible mechanisms of infection clearance (2, 5).

There are several historical case contact studies that point to the existence of a group of individuals who remain negative for reactivity to a TST despite heavy and repeated exposure to *Mtb* (2, 5, 6). These individuals have been previously labeled as “resisters” or “early clearers” of infection. For example, two different studies described the cumulative prevalence of these individuals among nurses and nursing students (7–9). One study took place at the Boston City Hospital from 1932 to 1947 and revealed that 52 out of 362 nurses (14.4%) fit this phenotype over a three-year follow-up period (7). The other study was performed at Fairview Hospital in Minneapolis and found that 16 out of 184 nursing students (8.7%) did not show any evidence of reactivity to a TST during a three-year follow-up (8). Another study performed in 1966 aboard the U.S.S. Richard E. Byrd found that 7 crew members out of approximately 70 who shared the same berthing compartment as a symptomatic pulmonary TB sailor remained TST negative during a 6-month follow-up (10). As one of these studies points out, these individuals appeared to be “endowed with a very superior resistance to tuberculosis which destroyed the tubercle bacillus before it could establish a ‘beach head’ in the body” (7).

Although recent work suggests this phenotype may be best characterized immunologically (4), several longitudinal epidemiological studies have attempted to define this “resister” phenotype using IGRAs in addition to the TST. Unfortunately, due to the heterogeneity of these studies as well as the instability observed in TST and IGRA results, a unifying definition has been difficult to ascertain.

Perhaps the greatest obstacle in trying to define a unified phenotype of resistance to TB is the inability to define a singular state. Resistance to *Mtb* remains a spectrum and is dependent on host, environmental and bacterial factors. To date, both household contact studies as well as community studies have been used to facilitate the identification of the spectrum of phenotypes of interest and these studies include cross sectional, case-control, and cohort study designs.

The purpose of this review is to, according to study design, summarize how different studies in the literature describe individuals who test IGRA and TST negative after single or multiple *Mtb* exposure (**Tables 1** and **2**). Second, we compare the differences. Third, based on the differences, we argue why a single definition of a “resister” phenotype may be inadequate. Lastly, we aim to make recommendations on how future studies should approach their study design and definitions.

**Table 1:** Summary of tests and definitions used by household contact studies.

Household contact study	Study location	Risk score used	Tests used	Cut-offs	Resister’ definition	Other definitions	LTBI definition
Stein et al. (11, 12)	Kampala, Uganda	Validated TB risk score by Mandalakas et al. (13); Ma et al. (14)	TST (5 tuberculin units [TU], PPD-S2, Tubersol, Sanofi Pasteur Limited, USA) QFT Gold In-Tube (QFT-GIT)	TST: 10mm (HIV− contacts) and 5mm (HIV+ contacts) QFT: IFN-γ (TB Ag – Nil) at 0.35 IU/ml	*Initial houlsehold contact study*: TST negative after at least 12 months of follow-up. *Retracing study:* Three negative QFT tests (one at baseline and two others during the 1-2 year follow-up period) and a final negative TST following the last QFT test. *Average duration of time in between studies:* 9.5 years.	**Probable ’resister’:** one ‘low level positive’ QFT that disagreed with the other 4 data points. A low level positive included values of 0.35 IU/ml < IFN-γ (TB Ag – Nil) < 0.5 IU/ml. **Possible ’resister’**: TST induration ≤ 8mm or one QFT positive plus all other negative tests or a TST negative incomplete with 2 negative QFTs but missing the last visit of the study.	**Definite LTBI:** All TST and QFT +. **Definite converters:** Persistently TST negative in initial study who converted to 3+ QFT and a +TST on retracing.
Mave et al. (15)	Chennai and Pune, India	Validated TB risk score by Mandalakas et al. (13); Ma et al. (14)	TST (5 TU, PPD, SPAN/Arkray, India) QFT-GIT	TST: 5mm QFT: IFN-γ (TB Ag – Nil) at 0.35 IU/ml	HHCs with high TB exposure who were TST and QFT negative at baseline and up to 12 months following exposure (TST < 5mm and QFT IFN-γ (TB Ag – Nil) < 0.35 IU/mL).	**Persistent LTBI negative (pLTBI-):** TST and QFT negative at baseline and up to 12 months following exposure. **’Resisters’ with complete absence of response**: TST = 0mm and IFN-γ (TB Ag – Nil) < 0.01 IU/ml.	**LTBI:** At least one positive TST or QFT test. TST ≥ 5mm or QFT IFN-γ (TB Ag – Nil) ≥ 0.35 IU/mL.
Verrall et al. (16, 17)	Bandung, Indonesia	Score, derived using regression methods, was based on the index TB case’s sputum smear grade, the presence of cavities and the extent of the CXR disease	QFT- GIT	IFN-γ (TB Ag – Nil) at 0.35 IU/ml	**Persistently negative (Early clearers):** Persistently IFN-γ (TB Ag – Nil) < 0.35 IU/ml at baseline and at 14 weeks.		**Conversion:** IFN-γ (TB Ag – Nil) < 0.35 IU/ml at baseline to IFN-γ (TB Ag – Nil) > 0.35 IU/ml at 14 weeks.
Hill et al. (18)	Banjul, The Gambia	No risk score used	TST (2 TU, PPD RT23, Statens Serum Institut, Denmark) ELISPOT	TST: 10mm ELISPOT: For a positive ESAT-6/CFP-10 result it was necessary for at least one of the two pools of overlapping peptides to be positive. Phytohaemagglutinin wells were set to at least 150 SFU/well/2 x 10^5 above negative control wells. Negative control wells were required to have less than 20 SFU.	HHCs with high TB exposure: TST < 5mm at baseline and 18 months following exposure and negative ELISPOT at baseline, 3 months and 18 months following exposure.		**TST conversion**: Negative at baseline and ≥ 10 mm & an increase in induration ≥ 6 mm at 18 months. **Positive ELISPOT:** ESAT-6/CFP-10- at least one of the two pools of overlapping peptides positive. **ELISPOT conversion:** as a newly positive test, plus a change in the combined ESAT-6 and CFP-10 count (> negative control) ≥ 6 SFU/well/2x10^5 (30 SFU/million cells).
Coulter et al. (19)	The Gambia	Based on sleeping proximity to the index TB case and smear grade of the index TB case	in-house IGRA	Unknown	**Non-converters:** IGRA negative at baseline and at 6 months		**LTBI:** IGRA positive at baseline **IGRA converters:** IGRA negative at baseline and IGRA positive at 6 months
Medawar et al. (20)	The Gambia	HHCs sleeping in the same room as index TB case	QFT-GIT	Negative QFT: IFN-γ (TB Ag – Nil) ≤ 0.2 Positive QFT: IFN-γ (TB Ag – Nil) ≥ 0.7	**QFT nonconverters**: IGRA negative at baseline and at 6 months	**QFT reverters: IGRA positive at baseline and IGRA negative at 6 months**	**LTBI:** IGRA positive at baseline and at 6 months **IGRA converters:** IGRA negative at baseline and IGRA positive at 6 months
Weiner et al. (21)	The Gambia	Based on sleeping proximity to the index TB case and smear grade of the index TB case	*Cohort 1*: TST (2 TU, PPD RT23, Statens Serum Institut, Denmark) *Cohort 2:* QFT-GIT	TST: 10mm QFT: IFN-γ (TB Ag – Nil) at 0.35 IU/ml	*Cohort 1* **TST nonconverters:** TST = 0mm at baseline and at 3 months. *Cohort 2* **Nonconverters:** IFN-γ (TB Ag – Nil) < 0.35 IU/mL at baseline and at 6 months.		**Cohort 1: (TST converters)** 0 mm at baseline and converted to positive >10 mm by 3 months. **Cohort 2: (converters)** IFN-γ (TB Ag – Nil) < 0.35 IU/mL at baseline and IFN-γ (TB Ag – Nil) > 0.35 IU/mL at 6 months.
Aissa et al. (22) and Cobat et al. (23)	Val de Merne, Paris, France	Exposure measures included daytime and nighttime proximity to the index case, duration of exposure to the index case in number of days during the 3 months prior to the index case’s diagnosis, and the index case infectivity. Index case infectivity was assessed using the duration of cough before diagnosis, presence of cavitation and extent of disease on CXR, and bacillary density in sputum smears and culture	TST (2 TU, PPD RT23, Statens Serum Institut, Denmark)	Aissa et al.: 5mm Cobat et al.: 0mm	*Aissa et al*.: TST < 5mm at baseline (V1) and 8-12 weeks (V2) *Cobat et al.*: TST = 0mm at V2 and V2		**LTBI:** TST ≥ 10 mm (no prior BCG vaccination). TST ≥ 15 mm at V1 or V2 or converted from TST < 5mm (V1) to TST ≥ 10 mm at V2 (BCG-vaccinated contacts).
Quistrebert et al. (24)	Southern Vietnam	No risk score used	*Vienam cohort*: TST (5 TU, Tubertest, Sanofi Pasteur, France) and QFT-GIT *Val de Merne cohort*: in-house IGRA *South African cohort*: in-house IGRA	TST: 5mm QFT: IFN-γ (TB Ag – Nil) at 0.35 IU/ml	*Vietnam cohort* **Double negatives:** TST < 5mm and QFT-GIT IFN-γ (TB Ag – Nil) < 0.35 IU/mL *Val de Merne cohort (Aissa et al.)* **Negative**: (i) TST < 5 mm at both V1 and V2, ii) < 5 mm at V1, when only one visit was done **Uninfected subjects:** HHCs with a negative TST and a null IFN-γ production *South African cohort* **Uninfected**: TST < 5 mm and a null IFN-γ production		*Vietnam study* **Double positives:** TST ≥ 5mm and a positive QFT-GIT IFN-γ (TB Ag – Nil) ≥ 0.35 IU/mL. *French study* **Positive:** TST i) ≥ 5 mm at both V1 and V2, ii) < 5 mm at V1 and ≥ 10 mm at V2. **Infected subjects** as HHCs who presented both a positive TST and a positive IGRA result (IFN-γ production > 175 pg/mL) *South African cohort* **Infected:** Both positive TST and IGRA result (IFN-γ production > 20.9 pg/mL).
Chen et al. (25)	Shanghai, China	Based on shared air space with an individual with pulmonary TB in the household or other indoor setting for > 15 hr per week or > 180 hr total during an infectious period (an infectious period was defined as the interval from 3 months before collection of the first culture-positive sputum specimen or the date of onset of cough, whichever was longer, through 2 weeks after the initiation of appropriate anti-tuberculosis treatment)	ELISPOT (TS-SPOT; Beijing Jinhao, China)	Positive if either Panel Test (containing ESAT-6/CFP-10/Rv3615c peptides pool) showed at least six spot-forming cells (SFCs) more than the negative control when the negative control ≤5 SFCs; or if the number of spots in Panel Test was at least double the number in the negative control when the negative control >5 SFCs	**Resisters:** persons who were highly exposed to *Mtb* and were IGRA/TST negative without clinical syndromes of active TB infection at enrollment. However, no TST was performed		**LTBI:** persons who were highly exposed to Mtb and were IGRA/TST positive without clinical syndromes of active TB infection at enrollment. However, no TST was performed
Vorkas et al. (26)	Port-au-Prince, Haiti	Based on living in the same house as the active TB case for at least 1 month in the 6 months prior to diagnosis	QFT-GIT	Not specified	HHCs with IGRA negative result at enrollment		HHCs with IGRA positive result at enrollment

**Table 2:** Summary of tests and definitions used by community-based studies.

Community-based study	Study location	Risk score used	Tests used	Cut-offs	Resister’ definition	Other definitions	LTBI definition
Cobat et al. (27)	Cape Town, South Africa	None. Risk is inferred from community exposure in a high incidence environment.	TST (2 TU, PPD RT23, Statens Serum Institut, Denmark)	TST: 0mm	TST = 0mm at baseline only.		TST > 0mm at baseline only.
Gallant et al. (28)	Cape Town, South Africa	None. Risk is inferred from community exposure in a high incidence environment.	TST (2 TU, PPD RT23, Statens Serum Institut, Denmark) In-house IGRA	TST: 5mm In-house IGRA: 63 pg/mL	**Double negatives** at a single time point. TST < 5mm and IFN-γ response to BCG or PPD or ESAT-6 < 63 pg/mL.		TST ≥ 5mm, IFN-γ response to BCG or PPD or ESAT-6 > 63 pg/mL.
Kroon et al. (29)	Cape Town, South Africa	None. Risk is inferred from community exposure in a high incidence environment.	TST (2 TU, PPD RT23, Statens Serum Institut, Denmark; 5 TU, PPD-S2, Tubersol, Sanofi Pasteur Limited, USA) QFT Gold Plus (QFT-Plus)	TST: 5mm QFT-Plus: Criteria for negative QFT-plus result: 1) Nil ≤ 8.0, and 2) TB1 minus Nil <0.35 or ≥0.35 and <25% of Nil value, and 3) TB2 minus Nil <0.35 or 0.35 and <25% of Nil value, and 4) Mitogen minus Nil ≥ 0.5. Criteria for positive QFT-plus result either: 1) Nil ≤ 8.0, and 2) TB1 minus Nil ≥0.35 and ≥25% of Nil value, and 3) Any TB2 minus Nil 4) Any Mitogen minus Nil Or 1) Nil ≤ 8.0, and 2) Any TB1 minus Nil 3) TB2 minus Nil ≥0.35 and ≥25% of Nil value, 4) Any Mitogen minus Nil.	**HIV-1-infected persistently TB, tuberculin and IGRA negative (HITTIN)**: HIV+ persons who had experienced a period of very low CD4 counts, who had no symptoms or history of previous TB, had three consecutive negative IGRA readings, and a TST = 0mm.		**HIV-1-infected IGRA positive tuberculin positive (HIT)**: HIV+persons who had experienced a period of very low CD4 counts, who had no symptoms or history of previous TB with two consecutive positive IGRA results and a TST ≥ 5mm.
Mahomed et al. (30)	Worcester, South Africa	Risk was inferred from community exposure in a high incidence environment. A subset of participants reported current or prior household contact, mostly within three years of enrollment.	TST (2 TU, PPD RT23, Statens Serum Institut, Denmark) QFT-GIT	TST: 5mm QFT-GIT: IFN-γ (TB Ag – Nil) at 0.35 IU/ml	Focus of the study was on converters/incident TB cases. No definition of resister/nonconverter given.		**Converters**: IFN-γ (TB Ag – Nil) > 0.35 IU/ml and TST ≥ 5mm at baseline measurement. 50% had active follow-up (every 3 months), and 50% had passive follow-up (at 2 year visit).
Nemes et al. (31)	Worcester, South Africa	Risk was inferred from community exposure in a high incidence environment. A subset of participants reported current or prior household contact, mostly within three years of enrollment.	QFT-GIT	QFT-GIT: IFN-γ (TB Ag – Nil) at 0.2, 0.35 and 0.7 IU/ml	**Stringent nonconverters:** IFN-γ (TB Ag – Nil) < 0.2 IU/ml at baseline, day 360, and day 720.	**Stringent QFT persistent positives:** IFN-γ (TB Ag – Nil) > 0.7 IU/ml at baseline, day 360, and day 720. **“Uncertain” converters:** IFN-γ (TB Ag – Nil) < 0.35 IU/ml at baseline, and IFN-γ > 0.35 IU/ml at day 360, with at least one result within the uncertainty zone of 0.2-0.7 IU/ml.	**Stringent converters:** IFN-γ (TB Ag – Nil) < 0.2 at baseline and > 0.7 at day 360).
Andrews et al. (32)	Worcester, Ceres & Robertson, South Africa	None. Risk is inferred from community exposure in a high incidence environment.	QFT-GIT	QFT-GIT: IFN-γ (TB Ag – Nil) at 0.35 and 4.0 IU/ml	**Nonconverters:** IFN-γ (TB Ag – Nil) < 0.35 IU/ml at baseline, day 336 and end of study.		**Converters:** IFN-γ (TB Ag – Nil) >4.00 IU/ml at baseline, day 336 and end of study.
Simmons et al. (33)	North West Province, South Africa	None. Risk is inferred from work exposure in gold mines, a high incidence environment.	TST (2 TU, PPD RT23, Statens Serum Institut, Denmark) QFT-Plus	TST: 0mm and 5mm QFT-Plus: Both antigen tube readings of IFN-γ (TB Ag – Nil) at 0.35 IU/ml	**"Uninfected" :** Miners who had a negative QFT-Plus up to one year after baseline. A stricter definition was also used as those with a negative QFT-Plus and TST = 0 mm after one year of follow-up.		**"TB infected":** Miners who had a positive QFT-Plus up to one year after baseline. A stricter definition was also used as those with a postive QFT-Plus and a TST > 5mm after one year of follow up.
Li et al. (34)	Beijing, China	None. Risk inferred from working at Beijing Chest Hospital ≥3 years	ELISPOT (T.SPOTTB; Oxford Immunotec)	ELISPOT ≥24 spots for ESAT-6, CFP-10, or both	**“Highly exposed but uninfected” (HEBUI):** **Negative ELISPOT at baseline only**		**"Latent":** Positive ELISPOT at baseline only

## Study Designs and Definitions

### Household Contact Studies

The past few years has seen increased interest in using household contact studies in order to understand the pathophysiology of *Mtb* infection—including the host immune response—and to define “resistance” or “early clearance” to infection. Some of the advantages of this particular study design include the ability to recruit a highly exposed group of individuals who can be followed prospectively while collecting extensive epidemiological data (11). All of these aspects ensure that all stages of *Mtb* infection and disease can be captured allowing a more defined and robust phenotypic examination (11).

#### Kampala, Uganda

The study by Stein et al. is unique among household contact (HHC) studies in that it revisited a cohort that had been originally recruited between 2002 and 2012 in order to assess the robustness of the “resister” phenotype several years later (12). This follow-up study retraced 407 HIV negative participants who were at least 15 years of age at the start of the retracing study and who had been initially classified as “persistent TST negative” (negative TST results for a minimum of 12 months and up to 24 months optimally) or “TST negative incomplete” (negative TST results for less than 12 months). The average time in between recruitment for these studies was approximately 9.5 years (12).

In addition to the TST used in the previous study, Stein et al. added the use of the QuantiFERON TB Gold In-Tube (QFT) test to further refine the “resister” phenotype. There were three QFT tests performed per participant. The first one was done at baseline followed by two others done in the 1 to 2-year period after baseline. A final TST (5 tuberculin units [TU], PPD-S2, Tubersol, Sanofi Pasteur Limited, USA) was performed following the last QFT test. Based on the additional testing, participants were further grouped into the following “resister” categories: “definite resister”, “probable resister”, and “possible resister” (see **Table 1**). The investigators defined certainty level using quantitative values of the QFT and TST such that if values were close to the positive/negative thresholds, they had a lower level of certainty. The levels of certainty also incorporated missed visits for both IGRA and TST data. Stein et al. also highlighted the presence of 32 TST/QFT “discordant” individuals who had consistent TST results but opposite QFT tests results. At the end of the study, most (82.7%) of the retraced “persistent TST negatives” remained QFT and repeat TST negative (“resisters”) while 16.3% converted to LTBI and 1.0% were labelled as “discordant”. In addition, 91.7% of these “resisters” had a TST of 0 mm (12).

#### Chennai and Pune, India

Mave et al. recruited a total of 799 children, adolescents and adults who had been living in the same household of an adult pulmonary TB index case from two different sites in India (15). Follow-up included three QFT tests and three TSTs (5 TU, PPD, SPAN/Arkray, India) at baseline, at 4 months and at 12 months. The cut offs used for the QFT tests were those suggested by the manufacturer and they used the more stringent 5 mm. cut off for the TST on all subjects. They also classified HHCs using a TB risk score and defined high exposure as those adults with a score > 6 and children with a score > 5 (13, 14). Using these tests, the authors defined the following phenotypes: “persistent LTBI negative” (pLTBI-), “resisters”, and “resisters with a complete absence of response” (**Table 1**). By the end of the follow up period, 91.6% of all HHCs developed latent TB infection. Sixty-seven HHCs (8%) were classified as “pLTBI-” and 52 of these were further classified as “resisters”. Although none of these “resisters” had a complete absence of response to both tests, approximately half of them had no response to at least one of the tests. Finally, the authors mentioned that the two tests only had a 60% agreement but did not reveal how they dealt with discordant results.

#### Bandung, Indonesia

This HHC study by Verral et al. sought to outline characteristics and associated risk factors of “early clearers” of *Mtb* infection (16, 17, 35). The HHCs for this study were recruited as part of the TANDEM project and originated in Bandung, one of the largest urban centers in Indonesia (36). The authors enrolled 1,347 HHCs of pulmonary TB index cases who were at least 5 years of age. Unlike the previous HHC studies, the authors only used the QFT test and the follow-up period was much shorter. The authors also used a different measure to evaluate the extent of exposure of HHCs. This score, which they derived using regression methods, was based on the index case’s sputum smear grade, the presence of cavities and the extent of the CXR disease (16, 17, 35). It also included the HHC’s number of hours spent with the index case as well as the sleeping proximity. To categorize each HHC, Verral et al. used QFT tests at baseline and 14 weeks later. The cut offs used were those recommended by the manufacturer. The authors defined “early clearers” as those HHCs who had negative QFT results at the end of the follow-up period (**Table 1**) (16, 17, 35).

Of the 1347 HHCs enrolled in the study, 490 were QFT negative at baseline and qualified for a follow up test. Of these, 317 (64.7%) had a subsequent negative QFT result and were labeled “early clearers” and 116 (23.7%) had a positive QFT result and were labeled “converters”. The rest could not be reached for a repeat test, had indeterminate results, had active disease or unevaluated symptoms of TB. The authors point out that the rate of “early clearers” in this particular study (~25%) was similar to those found in the cohorts from Uganda (14%) and The Gambia (45%) (6, 18). “Early clearers” had lower measures of exposures and, along with converters, were younger than those who had a positive QFT test at baseline.

#### The Gambia

There are four HHC studies from The Gambia that provide insight in the natural progression of *Mtb* infection. The first study by Hill et al. took place in Banjul and used the ELISPOT test and TST to define phenotypes while the study by Weiner et al. utilized the QFT and TST (18, 21). Coulter et al. and Medawar et al., utilized QFT only (19, 20).

Hill et al. analyzed the test results of 558 HHCs who were at least 15 years of age. These HHCs underwent 3 ELISPOTs at baseline, at 3 months and at 18 months (16). In addition, all HHCs also underwent a TST (2 TU, PPD RT23, Statens Serum Institut, Denmark) at baseline and a “subcohort” of 196 consecutively recruited HHCs also underwent a repeat TST at 18 months. The authors used a more stringent cut off for the ELISPOT than is recommended by the manufacturer. A positive TST required an induration of at least 10 mm plus an increase in such induration of at least 6 mm. Using the ELISPOT, the authors identified 97 (17%) HHCs who had three negative results during the 18-month follow-up. In the “subcohort” of 196 HHCs who underwent both ELISPOT and TST testing, 27 (14%) had consistently negative results after 18 months (**Table 1**).

Coulter et al. recruited 31 household TB contacts of 10 active TB index cases and classified them as LTBI, IGRA converters or non-converters, based on an in-house IGRA taken at baseline and 6 months later (19). Whole blood was stimulated by PPD, ESAT-6 and CFP-10. No details are provided on the threshold for IFN-γ positivity. Ten participants (32%) tested baseline IGRA positive and were defined as LTBI. Eleven (35%) participants who tested IGRA negative at baseline and remained negative at follow-up were defined as IGRA non-converters. Ten (32%) IGRA converters, who tested baseline negative and converted to positive at 6 months were also included. Exposure was measured by the smear grade of the index patient as well as sleeping proximity to the index patient (19). In a second study the group selected HHC from another longitudinal HHC cohort study (20). High exposure was defined by sleeping proximity and only persons sleeping in the same room as the index case was included. HHCs were seen at baseline and 6 months later. Seventeen (25%) were defined as “QFT nonconverters” based on 2 negative readings, 14 (21%) as “QFT converter” based on a negative at baseline and positive after 6 months, 18 (27%) as “QFT reverter” based on positive at baseline and negative after 6 months and lastly 18 (27%) as “LTBI” based on 2 positive readings. A QFT was considered negative if IFN-γ (TB Ag – Nil) < 0.2 IU/ml and positive if IFN-γ (TB Ag – Nil) > 0.7 IU/ml, which avoided what they referred to as the “grey zone” (20). It is not clear whether the participants represented in these two studies originated from the same cohort.

The fourth and most recent study by Weiner et al. was a case-control study nested within the larger study of HHCs at Medical Research Council Unit The Gambia (21). The authors used the same TB exposure score as Coulter et al. (19, 21). Weiner et al. aimed to characterize the host transcriptomic, metabolic, and antibody responses to *Mtb* in “nonconverters” when compared to “converters”. To do so, they established two different cohorts. In cohort 1, “nonconverters” were defined as HHCs who had a TST (2 TU, PPD RT23, Statens Serum Institut, Denmark) result of 0 mm at baseline and at the 3-month follow-up. In cohort 2, “nonconverters” were defined as HHCs who had a negative QFT test result at baseline and at the 6-month follow-up (**Table 1**) (21). Unfortunately, the authors did not provide the cut-off values they used to determine a negative QFT test result, nor did they mention the number of “nonconverters” identified.

#### Val-de Marne, Paris, France

This cohort of HHCs was described in multiple substudies aimed at characterizing the genetics of a *Mtb* infection resistance phenotype. They lived with pulmonary TB index cases for 3 months prior to their TB diagnosis and were recruited between April 2004 and January 2009. Between April 2004 to December 2005, 325 index cases and 2009 HHCs were initially identified and described (22). Participants were seen for two visits. During the screening visit (V1) a TST (5 TU, Tubertest, Sanofi Pasteur, France) was administered and blood was taken for an in-house IGRA. A repeat TST was administered 8–12 weeks later during visit 2 (V2).

A negative TST was defined as a TST reading <5 mm at both V1 and V2 or a single reading < 5 mm if only one visit (V1) was completed. In contacts without prior BCG vaccination a positive TST reading was defined as ≥ 10 mm. A TST reading was considered positive in BCG-vaccinated persons if it was ≥ 15 mm at both V1 or V2 or < 5 mm at V1 and converted to ≥ 10 mm at V2 (22). The authors also collected exposure measures of HHCs. These included daytime and nighttime proximity to the index case, duration of exposure to the index case in days during the 3 months prior to the index case’s diagnosis, and the index case infectivity. Index case infectivity was assessed using the duration of cough before diagnosis, presence of cavitation and extent of disease on CXR, and bacillary density in sputum smears and culture (22, 24).

Aissa et al. identified 1575 contacts who completed the screening process. Of these contacts a total of 1,150/1,575 (73%) remained uninfected, 410 (26%) had latent infection and 15 (1%) had active TB. Later and by using more stringent TST definitions, a total of 84/540 (15.6%) HHCs with TST readings of 0 mm at both visits, were defined and was used to replicate findings in the Sequella cohort which was recruited in South Africa and is described under community-based studies (**Table 1**) (23).

An additional in-house IGRA was later described in the same cohort (24, 37). The in-house IGRA was defined to measure IFN-γ production after stimulating peripheral blood mononuclear cells (PBMCs) with ESAT-6 and null production of IFN-γ was defined as a negative IGRA. A positive IGRA result was defined as IFN-γ > 175 pg/mL (24). A negative TST was defined as a TST reading <5 mm at both V1 and V2 or a single reading <5 mm if only one visit (V1) was completed. A TST reading was considered positive if it was ≥ 5 mm at both V1 and V2 or < 5 mm at V1 and converted to ≥ 10 mm at V2 (24).

Contacts were defined as *Mtb* infection “resisters” if they had a negative TST and null IFN-γ production, irrespective of previous BCG status. In total, 33/664 (5%) were identified as *Mtb* uninfected (TST negative and IGRA null). There were 147/664 (22%) infected (TST positive and IGRA positive) persons and 484 HHC who had discordant results or missing information. This cohort was used to validate loci identified in the HHC study in Vietnam (22, 24).

#### Southern Vietnam

This study included 1,108 HHCs of 466 pulmonary TB cases from 2010 to 2015 in an endemic region of Southern Vietnam (24). The objective of the study was to characterize the genetics of a TST and QFT negative *Mtb* resister phenotype. However, participants were not followed or defined longitudinally. Participants were invited for a baseline TST (5 TU, Tubertest, Sanofi Pasteur, France) and QFT test. A negative TST was defined as a TST reading < 5mm. QFT results were defined according to the manufacturer’s instruction (24). Although the authors defined the HHC subjects included in the study as being at high risk of infection, they did not provide information on any measures of exposure that they may have used to make this determination.

The study defined resistance to *Mtb* infection as a negative TST and IGRA reading at a single time point (“double negatives”). This group was compared to the group classified as *Mtb* infected i.e. a positive TST and IGRA reading (“double positives”). In total, 188 (17%) “double negatives” and 512 (46%) “double positives” were identified, as well as 408 participants with discordant results who were excluded (24).

#### Shanghai, China

The cross-sectional study by Chen et al. sought to identify the CD69 expression profiles of a number of different phenotypes, including “resisters” (25). The authors defined “resisters” as individuals who were close contacts to TB index cases with persistently negative TST/IGRA results despite prolonged exposure. Prolonged exposure was defined as sharing air space with an individual with pulmonary TB in the household or other indoor setting for > 15 h per week or > 180 h total during an infectious period. The infectious period was defined as the interval from 3 months before collection of the first culture-positive sputum specimen or the date of onset of cough, whichever was longer, through 2 weeks after the initiation of appropriate TB treatment. However, the authors only used the ELISPOT assay at baseline (according to the manufacturer’s instructions) and did not confirm the persistence of this result with subsequent tests. Based on these definitions, Chen et al. identified 13 “resisters” (25).

#### Port-au-Prince, Haiti

A cross-sectional study design was utilized to recruit HHCs from high transmission risk households (26). High risk of exposure of the HHCs was defined as sleeping in the same house as the TB index case for at least one month during the six months prior to the index case diagnosis. HHCs were seen at baseline and 6 months later and were screened for LTBI using QFT. Unfortunately, the authors did not provide the QFT cut-offs used in their study. At baseline, 19 (61%) HHCs tested QFT positive and 12 (39%) tested QFT negative. All of the twelve (39%) initially negative HHC remained IGRA negative on both visits and they were labeled as “TB healthy household contacts” who had “resisted infection” (26).

### Community Studies

In high TB burden settings, contact outside the household accounts for the majority of TB transmission in these settings. This occurs especially in cases of prolonged stay in low socio-economic communities with a high burden of TB and HIV (38, 39). Some activities associated with transmission include drinking in social groups, using public transportation, school and workplace exposures (38–46). Individuals who are severely immunocompromised, as with HIV-infection, are more susceptible to progress to TB (47–49).

Genotype and phenotypic drug susceptibility testing in the Western Cape, South Africa, show that considerable community transmission occurs in children <13 years with household TB transmission cases only contributing to around 8–19% (46, 50–53). Age can be used as a proxy for exposure frequency with at least 80% of individuals converting to positive TST reactions by the age of 30 (28, 45). High *Mtb* infection transmission rates in a high burden community show the importance of utilizing community-based research in these settings (45, 54, 55).

#### Sequella Study, Cape Town, South Africa

The Sequella study recruited 475 healthy, HIV-uninfected children and adolescents from 155 nuclear families from local clinics in two suburbs in Cape Town, South Africa (28). Blood was collected for an in-house IGRA and TST (2 TU, PPD RT23, Statens Serum Institut, Denmark) at baseline only. TST measurements were recorded as negative if TST < 5 mm. IFN-γ was measured after whole blood was stimulated with live BCG, PPD or ESAT-6 and positive responders were initially defined as IFN-γ responses of > 62 pg/mL (28).

The resister phenotype was defined as participants with double negative results at a single time point and were not longitudinally followed. A total of 164 (38%) were classified as TST negative or TST < 5 mm with 162 having TST readings of 0 mm and 260 with readings ≥ 5 mm. The double negatives identified were as follows: BCG negative (n=15), PPD negative (n=26) and ESAT-6 negative (n=81) (28).

More recently this cohort was used to validate findings in the aforementioned Vietnamese cohort (24). *Mtb* infection resisters were now defined as uninfected subjects with a negative TST (< 5 mm) and a null IFN-γ production [128/415 (31%)], and infected subjects as those with both positive TST and IGRA result (IFN-γ production > 20.9 pg/mL) [152/415 (37%)]. A third of the participants [135/415 (33%)] had discordant results.

#### Cape Town, South Africa

The ResisTB study is a community based case-control study conducted in Cape Town, South Africa (29). Participants were recruited from ART clubs at HIV clinics in Cape Town.

The “resistance” phenotype was defined as HIV-1-infected persistently TB, tuberculin and IGRA negative (HITTIN). All participants had to be HIV positive persons aged 35 to 60 years and living in an area of high transmission of *Mtb*, i.e., Cape Town. In addition, the criteria included a history of living with a low CD4+ count (either with two CD4+ < 350 cells/mm^3^ counts at least 6 months apart or a single CD4+ count < 200 cells/mm^3^) prior to initiating ART. During this period, these individuals would have been extremely susceptible to infection and disease. By the time of enrollment all participants were immune reconstituted on ART for at least one year with the most recent CD4+ count > 200 cells/mm^3^ (29).

Participants were screened with a QuantiFERON-TB Gold Plus (QFT-Plus) in-tube test and were classified as IGRA positive or negative according to the manufacturer’s instructions. Once identified participants were longitudinally followed-up. Individuals who tested IGRA negative were re-contacted on average 203 ± 151 days later for a second IGRA and TST administration (5 TU, PPD-S2, Tubersol, Sanofi Pasteur Limited, USA; 2 TU, Tuberculin PPD RT23, Statens Serum Institute, Denmark). The TST was read 3 days later and after this was done, a third IGRA was taken. Individuals in the final case group were designated HITTIN if they had three consecutive, negative IGRA tests and a negative TST reading (n=48) (**Table 2**). In parallel, a subset of control participants with an initial positive IGRA test were re-contacted for a second IGRA after an average of 292 ± 70 days. Those participants who tested IGRA positive in two consecutive tests (IGRA double+) and displayed a TST > 5 mm are defined as HIV-1-infected IGRA positive tuberculin positive (HIT, n=35) (29).

#### Worcester, South Africa

An adolescent youth cohort was recruited from local schools in Worcester, Western Cape, South Africa, during May 2005 until April 2007 (30, 31, 56–58). The TB notification rate in Worcester, was 1,400 cases per 100,000 in 1996. In total 6,363 adolescents aged 12–18 years (median 15yr, IQR:14–16) were enrolled into the cohort. The study included a majority younger participants ≤ 15 years old [56.5% (3603/6363)] and females [54.3% (3458/6363)] (58). Most of the participants, 1,055 of the 1,728 who had a current and prior household contact, reported the contact was within three years of the enrolment. Participants were screened with baseline TST (2 TU, Tuberculin PPD RT23, Statens Serum Institute, Denmark)) and QFT.

Nemes et al. investigated the consistency of serial QFT testing algorithms and included a more refined QFT conversion definition [a decrease from the manufacturer’s guidelines of IFN-γ (TB Ag – Nil) < 0.35 IU/ml to < 0.2 IU/ml and an increase from IFN-γ (TB Ag – Nil) > 0.35 to >0.7 IU/ml] to control technical and immunological variability that may occur within the “uncertainty zone” of 0.2–0.7 IU/ml (31).

The QFT results for participants in cohort 1 were classified according to the more stringent cutoffs compared to the manufacturer’s guidelines and were grouped into four categories. Stringent QFT nonconverters were defined as IFN-γ (TB Ag – Nil) < 0.2 IU/ml at baseline, day 360, and day 720. Stringent QFT persistent positives were defined as IFN-γ (TB Ag – Nil) > 0.7 IU/ml at baseline, day 360, and day 720. Stringent QFT converters were defined as IFN-γ (TB Ag – Nil) < 0.2 IU/ml at baseline and IFN-γ > 0.7 IU/ml at day 360. Lastly “uncertain” converters were defined as IFN-γ (TB Ag – Nil) < 0.35 IU/ml at baseline, and IFN-γ > 0.35 IU/ml at day 360, with at least one result within the uncertainty zone of 0.2–0.7 IU/ml (**Table 2**). A total of n=648/2,432 individuals were identified as stringent nonconverters and 989/2,432 were stringent persistent positives (31).

Applying a more stringent cutoff for a negative QFT result in cohort 1 improved concordance between TST and IGRA results. In the group with a negative QFT reading between 0.2–0.34 IU/ml, 53% had a discordant positive TST result, compared to 15% in the group with QFT IFN-γ values <0.2 IU/ml. In total 43% of those with QFT IFN-γ values between 0.2–0.7 IU/ml had discordant TST and QFT results, with 85% concordance in those with values < 0.2 IU/ml and > 0.7 IU/ml (31).

Importantly, stringent QFT nonconverters in cohort 1 had lower risk of developing TB disease (TB incidence 0.16 cases/100 Person-Years) than stringent QFT converters (TB incidence 1.60 cases/100 Person-Years, p=0.0003) and stringent persistent positives (TB incidence 0.97 cases/100 Person-Years, p=0.005). Due to immunological and technical assay variability “uncertain” QFT converters likely have a higher number of false positive converters since this group does not have a significantly different risk of TB disease compared to stringent nonconverters (TB incidence 0.66 cases/100 Person-Years, p=0.229) (31).

#### Rural Western Cape (Ceres, Robertson, Worcester), South Africa

Participants were recruited to a MVA85A tuberculosis vaccine trial during 2009 to 2011 in rural Western Cape, South Africa (32). The trial enrolled young children between 18–24 weeks old, with a median age of 20.4 weeks (IQR 19.3–22.0).

All children were screened with a baseline QFT. Similarly, to the previously described study stricter QFT cut-off values were applied (**Table 2**). A revised positive QFT test was defined as a IFN-γ (TB Ag – Nil) >4.00 IU/ml and a negative QFT as <0.35 IU/ml. Conversion was defined as a baseline negative QFT which was followed by a positive QFT. They defined an “uncertainty zone” of a QFT reading between 0.35–4.00 IU/ml. In total 2772/2797 of the children had a baseline negative QFT result. After 336 days, 2,512/2,772 (91%) had a repeat QFT and 2,327 (93%) remained QFT negative. QFT converters had higher risk of developing TB disease (TB incidence 28.0 cases/100 Person-Years) compared to those in the “uncertainty zone” (IRR 11.4; p=0.00047) and QFT non-converters (IRR 42·5; p<0.0001) (32).

This study did not use TST in conjunction with the QFT test. The generalizability is limited to young infants only, and because of young age, they likely have not been exposed to prolonged and sufficient *Mtb* exposure. Infants who developed active disease by day 336 were not included in the analysis and those who converted were given IPT. The authors suggest that QFT IFN-γ values ≥ 4.00 IU/ml in young children should prompt increased clinical diagnostic vigilance and potential interventions to prevent TB.

#### Gold Mines, South Africa

In South African gold mines, 13% of HIV-uninfected and 45.5% of HIV- infected gold miners tested TST = 0 mm (59). Due to a very high *Mtb* infection pressure and TB transmission in gold mines, transmission modelling assumes at least one lifetime infection in all gold miners (60). A study of goldminers, describes the long term follow up of some of these miners (33, 59, 61). Briefly, Simmons et al. analyzed a subset of 307 miners who were HIV-negative and had at least 15 years of mining experience in order to ascertain epidemiological factors associated with resistance to infection (**Table 2**). Both the QFT-Plus and TST (2 TU, PPD RT23, Statens Serum Institut, Denmark) were used during a one year follow up. The authors defined miners who were “uninfected” as those who had a negative QFT- Plus at baseline and one year later. They also used a stricter definition of “uninfected” as those miners who had a negative QFT- Plus and a TST = 0 mm. at baseline and one year later. Based on the stricter definition, the authors found that 18.7% of miners of Black/African ethnicity included in this analysis remained “uninfected” using the stricter definition. There is likely a spectrum of “resistance” to *Mtb* infection and given a high enough *Mtb* infection pressure, as in gold mines, most individuals are likely to become infected (2).

#### Beijing, China

A group of healthcare workers (HCW) in a TB hospital in Beijing, China were screened for Mtb infection with ELISPOT (T.SPOTTB; Oxford Immunotec) (34). A test was considered positive if ELISPOT ≥24 spots for ESAT-6, CFP-10, or both. HCW were only screened once at baseline. In total, 24 (50%) of HCW were defined as “latently” infected with a positive ELISPOT. The other half, 24 (50%) tested ELISPOT negative and were defined as “highly exposed but uninfected” (HEBUI). These HCW were enrolled if they had been working at the hospital for more than 3 years. Their work was considered high risk since standard infection control procedures such as wearing masks are not mandated, nor always followed (34).

## Discussion

Making comparisons across studies that have differing definitions of clinical groups of interest is a difficult proposition. A brief overview of the twenty studies presented in this review revealed seventeen different definitions for resistance to *Mtb* infection as measured by IGRA and TST. The definitions vary in a number of important categories, which include how the intensity and duration of exposure to *Mtb* was measured, the type of diagnostic tests and cut-offs used and the durability of the phenotype across different lengths of studies.

### Extent of Exposure

One of the important aspects of a HHC study is the opportunity to characterize the extent of exposure to *Mtb.* Unfortunately, only a minority of the HHC studies reviewed provided a good measure of this important factor. Stein et al. and Mave et al. utilized the same epidemiological risk score (11, 12, 15). This score is composed of a number of questions that provides a good understanding of the extent of exposure (i.e. whether the HHC share the same room or bed as the index case, whether the index case is actively coughing, whether the index case has a smear-positive sputum, whether the index case see the HHC every day) (11, 12, 15). The score can be used in both adult and pediatric populations. The use of this risk score allowed Stein et al. to make sure the extent of exposure would not differ between “resisters” and “converters” while Mave et al. included the score itself in their definition of resistance. Verral et al. also used an epidemiological risk score created specifically for their analysis using a logistic regression of exposure variables against QFT results. The resulting variable took into account both the intensity and duration of exposure (35). Of the studies that took place in The Gambia, Hill et al. did not use a risk score. On the other hand, Weiner et al. and Coulter et al. used a basic score composed of two variables (smear grade of the index case and the sleeping proximity to the index case), which they used to identify “nonconverters” and “converters” with the highest level of exposure (19, 21). Medawar et al. included HHC who were highly exposed based on sleeping in the same bedroom as the TB index case only (20). In another study, Vorkas et al. defined risk as a HHC who was sleeping in the same house as the TB case for at least a month during the 6 months before the index case was diagnosed (26). Finally, Chen et al. defined prolonged exposure as sharing air space with an individual with pulmonary TB in the household or other indoor setting for > 15 h per week or > 180 h total during a specific infectious period (25). The rest of the HHC studies, Cobalt et al., Jabot-Hanin et al., and Quistrebert et al., did not use a formal risk assessment of their participants as they concluded being a household contact living in the same residence as the index case would be enough to consider them at high risk of exposure. However, a household contact with a negative TST and/or QFT who qualifies for a study’s definition of “resister” may simply be the result of an exposure which is low in intensity or short in duration (2). This is why it is important to establish a measure of exposure, such as a validated epidemiological risk score, that could be used across studies.

The intensity of *Mtb* exposure is difficult to define within the context of a community based study design. Participants are usually unknowingly exposed to TB cases in comparison to HHC studies where there are defined TB cases and contacts. In high TB burden communities, the intensity of *Mtb* exposure is therefore inferred from community based rather than household transmission.

The extent of *Mtb* exposure is mostly defined by the duration of exposure in the community based studies. Incorporating an epidemiological risk score could be useful to identify and quantify possible high risk activities participants could be involved with e.g. classifying the amount of time an individual works in a high risk environment as with the gold mine studies, time spent using public transport in high incidence settings, previous or current TB contact and living in overcrowded conditions. Assigning risk scores to these activities would be cumbersome and difficult to substantiate, since none of these factors operate independently. For community based studies cumulative exposure to *Mtb* occurs by working or living in a high TB incidence environment (62). Simmons et al. defined a group of HIV-uninfected miners who worked for a prolonged time (>15 years) in South African gold mines which are known to be high TB risk environments (33, 59, 61). They restricted their analysis to include African miners who were more at risk based on poor socioeconomic status, living in crowded hostels and working underground in more poorly ventilated areas. Li et al. included HCW who worked for more than 3 years in a TB hospital where mask wearing was not mandated (34). In comparison, the ResisTB, Sequella and the Worcester based studies recruited participants from known high TB incidence areas (28, 29, 58). In addition, the ResisTB study used age as a proxy for exposure frequency and duration with older age (ages 35–60 years) representing a group who would have prolonged exposure in a high TB incidence environment (29). The results obtained from these community based studies are specific to the community described and one should be wary of making generalized conclusions.

### Diagnostic Tests

The “resister” definition should include both a negative TST and IGRA test result. The predictive value of using both tests is highlighted by the Mahomed et al. cohort which showed that TB incidence rates were higher for those participants with a baseline positive TST (≥ 5 mm) and IGRA (> 0.35 IU/ml) [0.6 cases per 100 person years (95% CI 0.43–0.82), 0.64 cases per 100 person years (0.45–0.87)], compared to participants who had baseline negative TST and IGRA results [0.22 cases per 100 person years (0.11–0.39), 0.22 cases per 100 person years (0.12–0.38)] (30). Of the studies reviewed, eight of them used both of these tests in their definitions of resistance (12, 15, 18, 24, 28–30, 57), eight of them only used an IGRA test (16, 17, 19, 20, 25, 26, 31, 32, 34, 35) and three of them only used a TST (23, 24, 27). Although Weiner et al. used both tests, they applied the TST to one cohort and the IGRA test to another cohort (21) (**Tables 1** and **2**).

A TST is a highly sensitive test and is a marker of TB immunoreactivity rather than a marker of infection (63). It is less specific than an IGRA and is known to have decreased specificity with false positives and cross-reactions to previous BCG vaccination and nontuberculous mycobacteria (NTM). Individuals with a negative TST may not have been sufficiently exposed to *Mtb*, or they were exposed but cleared infection. This could be either due to their own immunity or after receiving *Mtb* sterilizing prophylactic therapy such as Isoniazid. It is therefore imperative that documentation of *Mtb* exposure is maximized in the resistance phenotype as persons with prolonged exposure are less likely to revert and tend to remain TST positive after isoniazid preventive therapy, or even after completing TB treatment (63–66). However, application or reading errors, in addition to immunosuppressed states such as HIV-infection, immunosuppressive drug treatment and malnutrition could also account for false negatives.

Phenotypes defined as “resisters” or so-called persistently TST negative likely contain heterogeneous subgroups as discussed above. To improve the specificity of the phenotype, a TST is combined with an IGRA test. TST and *in vitro* IFN-γ assays do not measure similar aspects of host immunity and may depend on the host as well as the frequency and exposure setting of *Mtb* and NTM (28). In general, the proportion of individuals testing IGRA positive is lower than those having a positive TST and combining the two tests gives a stricter resistance definition (67).

There are two forms of commercially based IGRA tests available. One is based on an enzyme-linked immunosorbent assay (ELISA) and the other is an enzyme-linked immunosorbent spot (ELISPOT) assay. IGRA performs better as a marker of *Mtb* infection in a high compared to a low TB burden community and shows comparable reversion and conversion rates to TST (56). The studies covered in this review used different IGRA tests in their work. Galant et al. and the Val de Merne cohort described by Jabot-Hanin et al. and Quistrebert et al., as well as Coulter et al. described in-house IGRAs (19, 24, 28, 37). Hill et al., Chen et al. and Li et al. used an ELISPOT and the rest, except for Kroon et al. and Simmons et al. who used QFT-Plus, used QFT in their studies (18, 25, 29, 34). The latest QFT-Plus removed TB-7.7 from the assay and added a TB antigen tube with peptides which measure CD8+ cytotoxic T lymphocyte responses (68). This assay was developed to improve the lack of sensitivity of the QFT test, especially in HIV-infected persons. However, more studies are still needed to show that this is indeed the case (69). QFT-Plus shows good agreement with QFT (70–73) with mostly similar specificity and sensitivity (74–76). In addition, good concordance is seen between QFT, QFT-Plus and T.SPOT (73, 77).

IGRA results should be interpreted within the scope of performing the tests in low vs high TB burden settings, the immunocompetency of the patient and the range of output values from the tests (75, 78, 79). Output values often fall within a zone of uncertainty, defined as the total IFN-γ reading between 0.2–0.7 IU/ml after subtracting nil from TB Ag readings (31). Most participants with reversions tend to fall in this zone (31). This has been seen in QFT as well as QFT-Plus (73). Values falling in this zone are usually related to host immunological or technical variability (56, 80). This could also be indicative of participants who were recently infected and then possibly cleared infection. More reliable cut-offs for IGRA negativity set as values less than 0.2 IU/ml and > 0.7 IU/ml for a positive IGRA have been suggested (20, 31). Using these stricter definitions, they show that stringent nonconverters are less likely to develop TB over 2 years compared to recently converted or persistently QFT positive persons (31, 56). This would need to be evaluated within the context of the QFT-Plus assay which requires a value 25% greater than the nil value and a reading greater than the current standard cutoff of 0.35 IU/ml in either the TBAg1 or TBAg2 tube to be considered positive. Except for Nemes et al., all of the definitions for resistance in the literature use the established standard cutoffs when using IGRAs.

Finally, when using TST and IGRA tests, it is important to consider and collect information on factors that are associated with a positive result. For example, associations with smoking and diabetes have been established with positive TST and IGRA (81–84). Socio-economic factors such as low income and education, male sex, race, older age and HHC were identified as predictive factors for positive TST and IGRA results by Mahomed et al. (30). No significant differences were seen for socio-economic factors nor other factors such as smoking, BMI, and diabetes between HIT and HITTIN in the study by Kroon et al. (29). Mave et al. and Vorkas et al. also report no significant differences in demographic factors or clinical characteristics between converters and nonconverters (85). Simmons et al. report a BMI > 30 to be a risk factor for testing IGRA or TST positive (36). Similarly, Igo et al. found an association between a persistently TST negative result and a lower prevalence of lean mass wasting in the Uganda cohort. This is also in line with what some other studies have found (73, 86). Verrall et al. highlighted important evidence that BCG-vaccination provides dose dependent protection against IGRA conversion in HHC (16, 17). This protection effect is not seen in cases of high TB exposure and decreases with older age (16, 17, 87). In studies which show documented previous BCG vaccination or scars, no significant differences were seen between converters and nonconverters (12, 21), nor did a previous BCG show increased risk of conversion in Hill et al. (18). In addition, it would be of important for HHC studies to report the *Mtb* genotype as different strains are linked to TB clustering and variation in transmission (88). Verrall et al. reports an increased risk of conversion based on *Mtb* lineage, compared to Stein et al. who reported no difference in conversion based on *Mtb* lineage (12, 16, 17, 89).

### Durability of Responses

Based on previous epidemiological studies, and as suggested by Stein et al., resistance to *Mtb* does not appear to be absolute (12, 90, 91). In fact, there appears to be a threshold above which infection will be acquired due to a high enough intensity of exposure (2). Considering the shortcomings aforementioned of both the TST and IGRA tests, long term follow-up composed of multiple testing is imperative to evaluate the durability of the results. With an average follow up time of close to 10 years, Stein et al. provides sufficient time to evaluate their definition of resistance. This study also showed that the majority of conversions happened by month 3 of follow-up (9.8%), with 2.2% converting between 3–6 months and even still some (0.7%) converted after 6–12 months. Most of the individuals in this study remained both TST and QFT negative despite the long follow-up. Although it is not clear how the HHCs’ exposure varied in between the original and the follow-up study, since the participants continued living in a highly endemic area, we can presume that this did not change much. Based on these results, Stein et al. concluded that the “resister” phenotype is robust (12).

Similarly, the studies performed by Mave et al., Kroon et al., and Simmons et al. used both tests serially throughout a follow-up period of approximately year, which is an appropriate amount of time to capture conversions to LTBI or active disease (6, 15, 29). Of note, Mave et al. concluded that “pLTBI-” are rare and “resisters” are even rarer among HHCs. However, they attributed the low prevalence of these phenotypes partly to the more stringent cut-off use for the TST (15). Although not statistically significant, prevalence of the “pLTBI-” phenotype among children declined as age increased, which could certainly be the result of increased extent of exposure. Andrews et al. also had a sufficiently long follow-up of at least 336 days, however, the subjects were toddlers who were less than 2 years of age at enrollment (32). Both Nemes et al. and Mahomed et al. provided data on at least 2 years of either passive or active follow-up of adolescents with a maximum total time of 3.8 years (30, 31). A few studies followed participants between three to six months, a time when the majority of conversions would occur (23, 24, 26, 35, 37). The rest of the studies were cross-sectional in nature and only examined participants at a single time point (27, 28, 34). Despite their limitations, cross-sectional studies such as Cobat et al., Gallant et al., and Quistrebert et al., are appropriate to generate hypotheses and continue to contribute to our growing understanding of this phenotype. However, the durability and robustness of that which is measured remains unknown, since only a single time point is taken into account. Further follow-up to confirm robustness of the phenotype is needed to fully understand the implications of these biologic findings.

### Can a Unified “Resister” Definition Exist?

**Figure 1:**
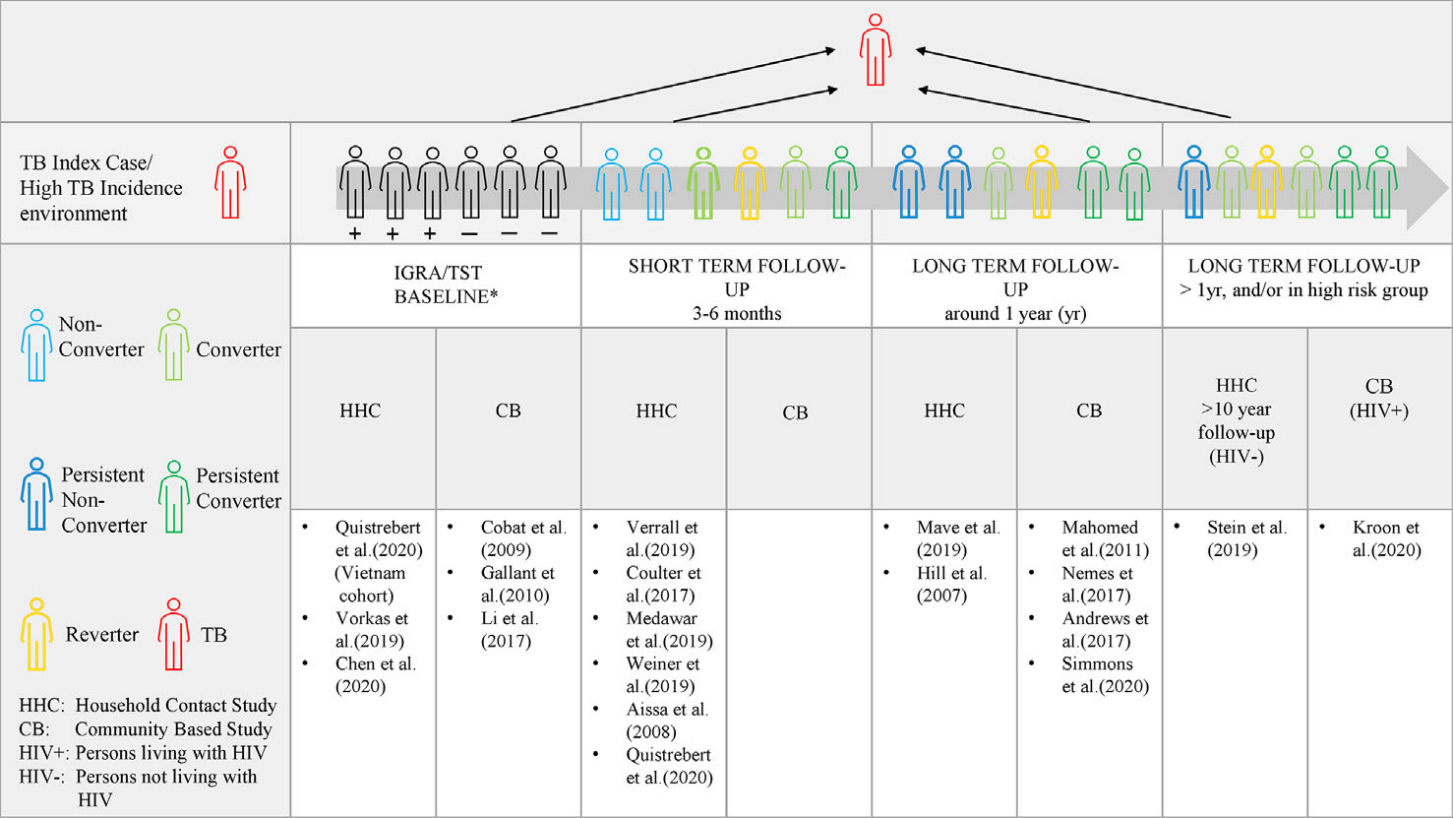
The spectrum of IGRA/TST nonconverters. Evidence of protection to *Mtb* at a shorter time period in relation to the index case, allows the study of correlates of protection which may be overcome by exposure intensity and frequency (represented by the grey arrow). Long term follow-up allows for a more robust and extreme phenotype definition as in Stein et al. (followed for 10 years) and Kroon et al. (in persons living with HIV). Persistent non-conversion is not absolute and may eventually be overcome by time and very high levels of exposure or continuous exposure. *At baseline, individuals may test either TST/IGRA positive, negative or be discordant.

Differences based on extent of exposure, diagnostic tests and durability of responses orders different studies according to a spectrum of host resistance (**Figure 1**). Early clearance is hypothesized to involve innate clearance of *Mtb* prior to an adaptive immune response (35). Theoretically, early clearance captures early response in a phenotype exposed to *Mtb* in an environment where *Mtb* exposure can be measured. “Early clearance” may not be achieved with each exposure and depends on the index case infectivity, TB severity and duration of contact (16, 17). In comparison, longitudinal studies with a longer follow-up, capture a more extreme and robust phenotype (12). The more extreme cases might only be captured by a smaller and high TB risk group, e.g persons living with HIV, who are able to remain TB free despite persistent or high intensity exposure (29). The entire spectrum is of value and contributes to our understanding host protection against *Mtb*.

Studies investigating adaptive response using additional immunological assays show IFN-γ independent T-cell responses, differences in T-cell receptors, as well as the presence of antibodies in many of the studies (4, 20, 21, 25, 29, 34). This highlights the significance of avoiding terms such as innate or adaptive resistance until both mechanisms have been further illustrated. Similarly, terms such as early clearance or resistance to *Mtb* infection are imprecise. Both assume infection but are unable to unequivocally prove it. Crucially, the description of what was measured should be given preference rather than using terminology inferring underlying immunological or biological events (92).

As has been previously proposed, descriptions of the observed states of outcome should rather be used (92). Based on Lalvani et al.’s suggestion and by using the easily accessible TST as well as IGRA at the baseline and follow-up visits, a phenotype can be defined as ‘persistently IGRA/TST negative (nonconverters), transiently IGRA/TST positive (reverters or conversion), or sustained IGRA/TST conversion (92)’. With prolonged follow-up and at least 2 visits, those who remain TST/IGRA negative are termed persistent nonconverters and those who remain positive would be termed persistent converters. For further definition, detailed innate and adaptive immunological assays are required (**Figures 1**).

## Conclusion

Based on our review, the lack of a unifying definition of the “resister” phenotype in the literature is apparent. As described above, we found differences in how some key components were assessed and incorporated into the definition of this phenotype. Moving forward, we propose the following recommendations when trying to identify “resisters” in household contact studies or community based studies: First, the extent of exposure must be measured and considered when defining resistance. This would be ideally done using an epidemiological risk score that has been validated across different types of studies and settings such as those used by Stein et al., Mave et al. and Verrall et al. Second, both IGRA and TST must be used when evaluating an individual’s response to *Mtb* and a stricter cut-off criteria should be considered for both tests. Third, the durability of the phenotype must be tested across multiple time points, ideally during at least a year of follow-up. Lastly, we propose that studies avoid terms which make assumptions about pathophysiological states which can only be inferred, such as infection. In addition, a review of both the innate and adaptive responses should be conducted before deciding on the final phenotype definition.

## Author Contributions

CS and MM conceived of the idea for the manuscript. JG and EK drafted the manuscript. All authors contributed to the article and approved the submitted version.

## Funding

This work was supported by the National Institutes of Health [1R01AI124349-01]. This research was partially funded by the South African government through the South African Medical Research Council (SAMRC) and supported by the National Research Foundation of South Africa. The content is solely the responsibility of the authors and does not necessarily represent the official views of the SAMRC. EK is supported through funding by the SAMRC through its Division of Research Capacity Development under the Clinician Researcher Development PHD Scholarship Programme. EK is also supported by a Career Development Fellowship [TMA2018CDF-2353-NeutroTB] awarded by The European and Developing Countries Clinical Trials Partnership. JG was supported by NIH training grant HL007567 and CS was supported by NIH grants R01AI124348, UO1-AI-09-001, and RO1AI147319. Funders were not involved in the writing of the manuscript or the decision to submit it for publication.

## Conflict of Interest

The authors declare that the research was conducted in the absence of any commercial or financial relationships that could be construed as a potential conflict of interest.
